# Anæsthesia by Compression

**Published:** 1858-11

**Authors:** 


*Anæsthesia by Compression.*—M. Jacowski, a practitioner of Paris, has revived the practice of compression as a means of preventing pain; and in several instances an entirely painless extraction of teeth have been effected. The compressor he employs consists of two pads, connected by a steel spring, very much like a hernial truss. The spring is passed behind the head, and the pads are applied either within the meatus auditorius on each side, or behind the rami of the jaw in front of the ears.—*Med. Times and Gazette,* from *Medical News and Library.*

